# The role of child behavioral inhibition in the intergenerational transmission of anxiety in early childhood

**DOI:** 10.1017/S0954579426101643

**Published:** 2026-07-09

**Authors:** Michelle Bosquet Enlow, Asja Abron, Dashiell Sacks, Caroline M. Kesley, Charles A. Nelson

**Affiliations:** 1 Department of Psychiatry and Behavioral Sciences, Boston Children’s Hospital, Boston, MA, USA; 2 Department of Psychiatry, Harvard Medical School, Boston, MA, USA; 3 Division of Developmental Medicine, Boston Children’s Hospital, Boston, MA, USA; 4 Department of Pediatrics, Harvard Medical School, Boston, MA, USA; 5 Harvard Graduate School of Education, Boston, MA, USA

**Keywords:** anxiety, behavioral inhibition, early childhood, intergenerational, temperament

## Abstract

Although the intergenerational transmission of anxiety is well documented, the underlying mechanisms remain elusive. In a community sample of mother–child dyads (*N* = 541; 54% male, 72% non-Hispanic White), we examined whether child behavioral inhibition (BI), a temperament characteristic that increases anxiety risk, mediated and/or moderated associations between maternal and child symptoms in early childhood. Greater maternal symptoms in infancy and at 3 years were associated with greater child symptoms at 3 years and 5 years (*r*s = .15–.23, *p*s < .01), but not with child BI at 3 years (*r*s = .00–.01, *p*s > .92). Thus, evidence for mediation was not observed. Moderation analyses revealed an interactive effect at 3 years (*β* = −0.13, *p* = .009): Among children with high BI, child symptoms were elevated regardless of maternal symptoms. Among children with moderate to low BI, greater maternal symptoms were associated with greater child symptoms. A similar pattern was observed at 5 years (*β* = −0.10, *p* = .113). Such associations were not observed when testing maternal depressive symptoms as predictor or child externalizing symptoms as outcome, supporting specificity for BI in intergenerational anxiety processes. Sex-specific effects in relation to BI were not observed. These findings contribute to our understanding of the role of child BI in the intergenerational transmission of anxiety in early childhood.

The intergenerational transmission of anxiety is a well-documented phenomenon, with children of parents with an anxiety disorder at a 2- to 4-fold increased risk of developing an anxiety disorder (Aktar & Pérez-Edgar, 2024; Lawrence et al., 2019; Micco et al., 2009). The elevated rates of anxiety found among children of affected parents indicate that these families may particularly benefit from targeted interventions. This is an urgent public health need, given that anxiety is the most common mental health condition affecting both children and adults, negatively impacting multiple domains of functioning and predicting lifelong mental health problems that are often refractory to treatment (Chiu et al., 2016; Cohen Kadosh et al., 2014; Kessler et al., 2012; Merikangas et al., 2010). Moreover, even nonclinical levels of anxiety beginning in early childhood are associated with behavioral and social problems that can persist throughout development (Mistry-Patel & Brooker, 2023). The design of effective interventions requires the identification of responsible underlying processes that can be targeted, ideally in preventative efforts initiated prior to the onset of child psychopathology in high-risk families.

Behavioral inhibition, a temperament characteristic defined by enhanced vigilance to novelty and lack of approach or active avoidance of unfamiliar objects, people, and environments (Fox et al., 2023), may play an important role in the intergenerational transmission of anxiety. Behavioral inhibition is the temperament trait most robustly associated with concurrent and later development of anxiety as well as with neural and physiological correlates of/risk factors for anxiety (e.g., elevated autonomic reactivity, attentional bias to threat, heightened neural responding to perceived self-made errors; Fox et al., 2023; Hudson et al., 2019; Kagan et al., 1987; Lorenzo et al., 2022; McDermott et al., 2009; Perez-Edgar et al., 2010; Tang et al., 2020). Although most commonly associated with measures of social anxiety, behavioral inhibition also has been associated with increased risk for separation anxiety, generalized anxiety, and anxiety symptoms and anxiety disorders more generally (Hudson & Dodd, 2012; Hudson et al., 2011). Notably, children of anxious parents are more likely to be behaviorally inhibited than children of non-anxious parents, and behaviorally inhibited children are more likely to have anxious parents (Aktar & Pérez-Edgar, 2024; Rosenbaum et al., 1993). Possible mechanisms linking parental anxiety and child behavioral inhibition include genetic and epigenetic factors, prenatal programming processes, and fear-reinforcing caregiving behaviors (Fox et al., 2023). Together, these findings suggest that behavioral inhibition may play a mediating role in the intergenerational transmission of anxiety, with parental anxiety increasing the likelihood of the child demonstrating heightened behavioral inhibition, which in turn increases the child’s risk for the development of anxiety problems. Longitudinal studies are needed to test these pathways beginning in early childhood, when these processes may first emerge and begin to stabilize (Aktar & Pérez-Edgar, 2024; Hudson et al., 2019).

Data also suggest that behavioral inhibition could play a moderating role in the intergenerational transmission of anxiety, although evidence for the specific nature of such effects is mixed (Aktar et al., 2013). Diathesis-stress and differential susceptibility models are possible candidates, with temperament being conceptualized as an individual, biologically-based risk (i.e., diathesis or susceptibility) factor that influences the child’s responses to exposure to parental anxiety (Aktar & Pérez-Edgar, 2024; Hudson et al., 2019; Lawrence et al., 2020). For example, behaviorally inhibited children may be more sensitive generally to environmental factors or specifically to signals of threat in their environment (Aktar & Pérez-Edgar, 2024; Zeytinoglu et al., 2025) and thus more likely than children who are not behaviorally inhibited to develop anxiety in anxious caregiving environments. Further, the stability for behavioral inhibition across development is modest, and the majority of behaviorally inhibited children do not develop an anxiety disorder (Clauss & Blackford, 2012; Fox et al., 2001, 2005, 2023, Price & Kiel, 2022). Caregiving behavior has been identified as one of the major moderating factors that influences whether a child remains behaviorally inhibited and develops anxiety (Fox et al., 2023; Lorenzo et al., 2022; Natsuaki et al., 2013). Compared to parents without anxiety, anxious parents are more likely to engage in behaviors that signal threat (e.g., modeling of fearful behavior, verbal and nonverbal communications of threat in the environment); behaviors that maintain their child’s avoidant and fearful behaviors (e.g., lack of encouragement to confront novel situations); and behaviors that undermine the development of their child’s emotion regulation abilities more generally (e.g., high levels of controlling and overprotective behavior) (Aktar & Pérez-Edgar, 2024; Fox et al., 2023; Murray et al., 2008; Murray et al., 2014). Moreover, there is evidence that such anxiogenic caregiving behaviors are exhibited more frequently by parents of behaviorally inhibited children, suggesting that behaviorally inhibited children may elicit such parenting behaviors (Murray et al., 2014; Raishevich et al., 2010). Finally, data support the hypothesis that behaviorally inhibited children are more likely to develop anxiety than children who are not behaviorally inhibited in the context of such caregiving behaviors (Aktar et al., 2013; Hudson et al., 2019; Lawrence et al., 2020; Murray et al., 2014). Thus, behavioral inhibition may increase child risk for anxiety specifically in the presence of parental anxiety, and exposure to parental anxiety may have particular impact on anxiety risk among behaviorally inhibited children (Hudson et al., 2019; Lawrence et al., 2020).

Alternatively, behavioral inhibition and parental anxiety may each having a ceiling effect on a child’s risk (Aktar & Pérez-Edgar, 2024). Consequently, parental anxiety may only increase risk for anxiety problems among children with low levels of behavioral inhibition; behaviorally inhibited children are already at elevated risk, which may not further increase in the presence of parental anxiety (Aktar & Pérez-Edgar, 2024; Blackford & Walden, 1998). Similarly, behavioral inhibition and parental anxiety may share or influence similar underlying biological mechanisms that affect child anxiety vulnerability (e.g., genetic factors, increased physiological stress reactivity, neural and behavioral responding to threat), such that having one or both risk factors confers similar child risk (Fox et al., 2023). Some studies support this hypothesized association, finding that children who show very high, stable levels of behavioral inhibition do not experience increased risk in the context of parental anxiety, whereas children who are not temperamentally at risk are more affected by parental anxiety (Aktar & Pérez-Edgar, 2024; Blackford & Walden, 1998).

Child behavioral inhibition also may play a role independent from that of parental anxiety in influencing child anxiety risk (Hudson et al., 2011; Shamir-Essakow et al., 2005). There is evidence that behavioral inhibition and parental anxiety may increase child risk through separate pathways, each independently contributing to child vulnerability, with children who are both behaviorally inhibited and have anxious parents at greatest risk for experiencing anxiety problems (Hudson et al., 2011; Shamir-Essakow et al., 2005). Additionally, there is evidence that parental anxiety similarly increases child anxiety risk among behaviorally inhibited and uninhibited children (Hudson et al., 2019).

Thus, the extant literature suggests a possible role of child behavioral inhibition in the intergenerational transmission of anxiety. However, as described above, study results to date do not point to a consistent model as to how child behavioral inhibition influences child vulnerability in the context of parental anxiety. Several methodological considerations may contribute to these mixed findings. For example, studies have varied as to when and how often they assess child behavioral inhibition; although behavioral inhibition is observable beginning in infancy, it shows moderate stability over time. Therefore, when in development behavioral inhibition is assessed, and whether it is assessed at a single timepoint or as a stable trait evident over multiple timepoints, may influence how it is associated with parental and child anxiety. Studies also vary in how behavioral inhibition is assessed (e.g., parental questionnaire, observational protocols in response to various types of tasks) and scored (e.g., categorical vs continuous), which may influence its predictive abilities. Studies have also varied in how they operationalize parental anxiety (e.g., presence/absence of lifetime anxiety disorder; number of current or lifetime anxiety disorders; continuous measures of anxiety symptoms; social anxiety disorder/symptoms vs other forms of anxiety) and child anxiety outcomes (internalizing symptoms, general/total anxiety symptoms, social anxiety symptoms/disorder). Thus, additional research is needed to understand how parental anxiety and child behavioral inhibition may jointly contribute to the intergenerational transmission of anxiety, with explicit consideration of when and how child behavioral inhibition and parental and child anxiety are assessed.

## The current study

**Figure 1.**
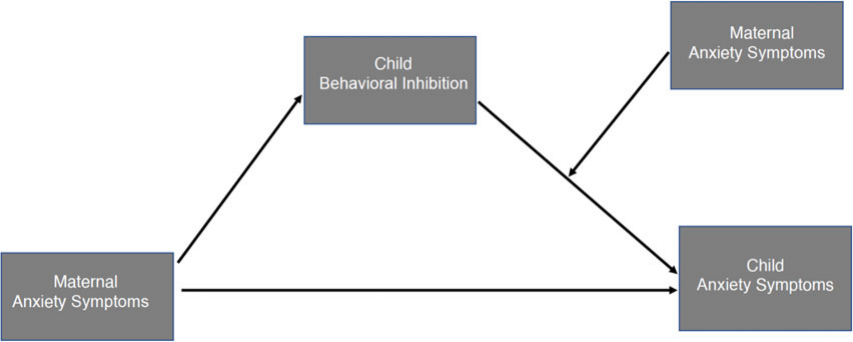
Conceptual model depicting child behavioral inhibition as a mediator and as a moderator of the association between maternal anxiety symptoms and child anxiety symptoms.

In this study, we examined the potential role of child behavioral inhibition in the intergenerational transmission of anxiety in early childhood, when behavioral inhibition is observable and child anxiety problems may begin to emerge (Hudson et al., 2019). Specifically, in a longitudinal community cohort of typically developing children, we tested whether maternal anxiety symptoms measured at infancy and 3 years were associated with child anxiety symptoms at ages 3 years and 5 years and whether child behavioral inhibition assessed at age 3 years mediated and/or moderated any observed associations between maternal and child symptoms (Figure 1).

We hypothesized that greater maternal anxiety symptoms would be associated concurrently and longitudinally with greater child anxiety symptoms, given well-documented evidence of increased anxiety risk among offspring of parents with anxiety (Aktar & Pérez-Edgar, 2024; Lawrence et al., 2019; Micco et al., 2009). We further hypothesized that higher child behavioral inhibition would mediate associations between greater maternal and child anxiety symptoms, supporting a direct association among variables, given evidence that parental anxiety increases likelihood of child behavioral inhibition and that child behavioral inhibition increases child risk for anxiety (Aktar & Pérez-Edgar, 2024; Rosenbaum et al., 1993). Additionally, we hypothesized that child behavioral inhibition would moderate the effects of maternal anxiety symptoms on child anxiety symptoms, supporting an interactive effect. As described above, the existing literature suggests the possibility of several models of joint effects, including that (a) maternal anxiety has a stronger influence on child anxiety risk among children higher in behavioral inhibition; (b) maternal anxiety has a stronger influence on child anxiety risk among children lower in behavioral inhibition, with children higher in behavioral inhibition at increased anxiety risk regardless of maternal anxiety status; and (c) maternal anxiety and child behavioral inhibition additively contribute to child anxiety risk, with children who are behaviorally inhibited and exposed to elevated maternal anxiety at greatest risk (Aktar & Pérez-Edgar, 2024; Aktar et al., 2013; Blackford & Walden, 1998; Fox et al., 2023; Hudson et al., 2019; Lawrence et al., 2020; Murray et al., 2014; Shamir-Essakow et al., 2005; Zeytinoglu et al., 2025). For the current study, we hypothesized that children with higher levels of behavioral inhibition would show a stronger association between maternal and child symptoms than children with lower levels of behavioral inhibition. We further hypothesized that the children at highest risk for anxiety problems would be those who had elevated scores on both behavioral inhibition and maternal anxiety symptoms.

We examined concurrent and longitudinal associations among maternal anxiety symptoms, child behavioral inhibition, and child anxiety symptoms, as it is still an open question as to how early in development we can identify children with behavioral inhibition who have an elevated risk for anxiety, how stable this prediction remains across childhood, and when in development parental anxiety may exert greatest impact on children temperamentally at risk (Aktar & Pérez-Edgar, 2024; Fox et al., 2023).

Finally, we conducted a series of secondary analyses to test for specificity. First, potential differential sex effects were explored, given evidence for sex differences in response to maternal psychopathology as well as the impact of behavioral inhibition on socioemotional functioning (Bale & Epperson, 2015; Fox et al., 2023; Murray et al., 2008). Second, to examine whether any significant findings were specific to the intergenerational transmission of anxiety, we conducted separate analyses replacing maternal anxiety symptoms with maternal depressive symptoms and child anxiety symptoms with child externalizing symptoms. Existing evidence for associations between maternal depression and child behavioral inhibition is mixed. Some studies suggest that both parental depression and anxiety are associated with increased child behavioral inhibition, whereas others suggest that the association is specific to anxiety and that findings linking parental depression to child behavioral inhibition are spurious correlations resulting from the high comorbidity between anxiety and mood disorders (Durbin et al., 2005; Fox et al., 2023). Analyses conducted with child externalizing symptoms as the outcome examined specificity of these processes to child anxiety problems, as prior literature suggests BI in early childhood specifically increases risk for internalizing problems, particularly anxiety, and not for externalizing problems or general psychopathology (Tang et al., 2020).

## Method

### Participants

Participants were recruited between 2012 and 2016 from a registry of local births comprising families who had indicated willingness to participate in developmental research. Families in the current analyses were enrolled in a prospective study to examine the early development of emotion processing. Exclusion criteria included known prenatal or perinatal complications, maternal use of medications during pregnancy that may have significant impact on fetal brain development (i.e., anticonvulsants, antipsychotics, opioids), pre- or post-term birth (≥ ± 3 weeks from due date), developmental delay, uncorrected vision difficulties, and neurological disorder or trauma. After enrollment, families were no longer followed and their data were excluded from analyses if the child was diagnosed with an autism spectrum disorder or a genetic or other condition known to influence neurodevelopment. By design, families were enrolled in the parent study in infancy, with a subsample followed through later assessments. To be included in the current analyses, participants needed to provide, at minimum, data from two of the three timepoints assessed (infancy, 3 years, 5 years), which provided an analytic sample of *N* = 541.

### Procedures

Mothers were asked to complete questionnaires via an online survey prior to each of the laboratory visits at infancy (*M*_age_ = 8.1 months, SD = 3.0 months), 3 years (*M*_age_ = 3.1 years, SD = 0.2 years), and 5 years (*M*_age_ = 5.2 years, SD = 0.2 years). Questionnaires relevant to the current analyses included assessments of sociodemographic characteristics (infancy), maternal anxiety symptoms and depressive symptoms (infancy, 3 years), and child symptoms (3 years, 5 years). During the 3-year laboratory visit, children participated in a standardized laboratory observational assessment of child behavioral inhibition. The Institutional Review Board at Boston Children’s Hospital approved all methods and procedures used in this study, and mothers provided written informed consent prior to the initiation of study activities.

### Measures

#### Sociodemographics

At infancy, the child’s mother completed online questionnaires that inquired about the child’s sex, age, and race/ethnicity, parental age and educational attainment, and annual household income.

#### Maternal anxiety symptoms (primary predictor)

Maternal anxiety symptoms were measured at infancy and 3 years via the Trait Anxiety form of the Spielberger State-Trait Anxiety Inventory (STAI-T; Spielberger et al., 1983). The STAI is a 20-item self-report questionnaire designed to measure anxiety proneness by asking the respondent to rate the frequency of general mood states on a 4-point scale, ranging from “almost never” to “almost always.” Item scores were summed to create a total score (possible range: 20–80), with higher scores indicating greater anxiety and scores ≥40 suggesting clinically relevant levels of anxiety (Grant et al., 2008; McMahon et al., 2001). The median alpha coefficient of internal consistency has been reported as 0.90 and test-retest coefficients from 0.73 to 0.86 (Barnes et al., 2002).

#### Behavioral inhibition (mediator, moderator)

Child behavioral inhibition (BI) was assessed using standardized batteries designed to measure children’s avoidance of novel contexts, objects, and social situations (Fox et al., 2023). At the 3-year visit, participants completed the behavioral tasks described below (Fox et al., 2001; Kochanska, 1995). At the start of the protocol, the mother and child entered the behavioral testing room, and the mother was seated in a corner of the room and given a pair of headphones to wear while completing a set of questionnaires. The mother was instructed not to initiate interactions or prompt the child to behave in any specific way and to respond minimally if their child went to them seeking comfort. The child’s responses were video recorded throughout the tasks.

*Stranger*. During the first task, an unfamiliar experimenter (“stranger”) entered the room. The stranger then sat in silence with their head down for one minute before taking out a toy truck and playing with it for another minute in silence. If the child did not approach the stranger to play, the stranger continued to play with the truck for one minute, prompting the child to play with them twice during that time using a neutral tone of voice. Other than during the prompts, the stranger did not make eye contact with the child.

*Robot*. The stranger then left the room with the truck and came back with a quadruped robot. The robot crawled unpredictably on the floor, making loud noises and flashing lights. The stranger prompted the child to play with the robot twice during the 2-minute period. Aside from during the verbal prompts, the stranger did not make eye contact with the child.

*Tunnel*. The stranger then removed the robot from the room and came back with a collapsible play tunnel. After unfolding the tunnel and placing it on the ground, the stranger prompted the child to crawl through the tunnel three separate times before removing the tunnel from the room. The stranger did not make eye contact with the child except during verbal prompts.

*Blood pressure cuff*. The stranger then asked the child to sit on a chair and try on a blood pressure cuff. The stranger delivered a maximum of four prompts for the child to present their arm to try on the cuff.

*Stairs*. The stranger then asked the child to climb to the top of a small set of steps and jump onto a mat. The stranger delivered a maximum of three prompts for the child to jump.

*Snake*. The stranger then opened an aquarium containing a plastic snake and asked the child to touch the snake. The stranger delivered a maximum of three prompts for the child to touch the snake.

*Black box*. The stranger then presented the child with a black box and asked the child to reach inside the black box. Inside the box was a stuffed animal, but the child was not told this information. The stranger delivered a maximum of six prompts for the child to reach inside the black box.

*Coding*. Heightened BI is suggested by slow approach behaviors and proximity seeking of the caregiver in response to novel tasks (Fox et al., 2023). Here, we coded three measures for each task: (1) latency to the target action (e.g., approached the stranger to play, touched the snake), (2) time spent near the mother, and (3) target action completed (yes, no). The BI composite score was calculated by averaging the *z*-scores of these three measures across all seven episodes and was considered in analyses as a continuous measure. Interrater reliability was assessed for 20% of the sample and showed high agreement between coders, with the inter-class correlations ranging from 0.78 to 1.00.

#### Child anxiety symptomatology (primary outcome)

*Infant-Toddler Social and Emotional Assessment (ITSEA)*. At 3 years, mothers completed the ITSEA, a 166-item questionnaire validated for the assessment of emotional and behavioral problems in children between 1 and 3 years of age (Carter et al., 2003; Carter et al., 2006). Mothers were asked to assess how often particular behaviors occurred on a 3-point scale (0 = rarely/not true, 1 = sometimes/somewhat true, 2 = often/very often true). For the current analyses, scores from two anxiety scales were utilized: anxiety/worry and separation distress. The 7-item anxiety/worry scale inquires about symptoms reflective of generalized anxiety and phobias. The 6-item separation distress scale inquires about symptoms reflective of separation anxiety. The anxiety/worry and separation distress scale scores were standardized and then averaged to create an anxiety symptom scale score at 3 years.

*Child Behavior Checklist 1.5–5 (CBCL/1.5–5)*. At the 5-year assessment, mothers completed the Child Behavior Checklist 1.5–5 (CBCL/1.5–5; Achenbach & Rescolar, 2000). The CBCL forms are among the most well-established, empirically supported questionnaires to assess child psychopathology symptoms (Achenbach et al., 2008). The 99-item CBCL/1.5–5 asks parents to report on their children’s behavior during the past six months on a 3-point scale (0 = not true, 1 = somewhat or sometimes true, 2 = very true or often true). For the current analyses, the raw score for the 10-item Anxiety Problems DSM-oriented scale was used. This scale inquires about symptoms of generalized anxiety/worries, phobias, and separation anxiety. Raw scores were chosen over *T*-scores to maintain the distribution of symptoms, as *T*-scores for CBCL symptom scales truncate *T*-scores < 50 to 50.

#### Maternal depressive symptoms (predictor, secondary analyses)

Maternal depressive symptoms were measured in infancy and at 3 years via the revised Beck Depression Inventory (BDI-IA; Beck & Steer, 1993; Beck et al., 1988), a 21-item, highly reliable (internal consistency estimates from 0.73 to 0.92; Dozois et al., 1998) and valid self-report questionnaire that assesses the frequency and intensity of depressive symptoms over the prior two weeks. Individual items were scored on a 4-point scale (range 0–3) and summed for a total possible range of 0–63. Higher scores indicate greater depression severity, with scores of 0–9 suggesting no or minimal depression, 10–18 mild depression, 19–29 moderate depression, and 30–63 severe depression (Beck et al., 1988).

#### Child externalizing symptoms (outcome, secondary analyses)

Child externalizing symptoms were measured at 3 years via the ITSEA broad-band Externalizing Problems scale (subscales: activity/impulsivity, aggression/defiance, peer aggression) and at age 5 years via the CBCL/1.5–5 Externalizing Problems scale (subscales: attention problems, aggressive behaviors). Raw scores were calibrated and normed by child age and sex, with normed scores expressed as the standard *T*-score metric (*M* = 50, *SD* = 10).

### Data analysis plan

Analyses were conducted using SPSS (IBM; version 28.0.1.0) and *R* (version 4.3.2). We first performed descriptive analyses to characterize the sample. Given potential sex differences in child BI and anxiety symptom scores, we examined whether these scores differed between male and female participants to inform whether sex should be included as a covariate in analyses.

To address our main aims, we analyzed associations among maternal anxiety symptoms in infancy and at 3 years, child behavioral inhibition at 3 years, and child anxiety symptoms at 3 years and 5 years. If correlation analyses supported potential mediation effects, i.e., if maternal anxiety symptoms were associated with child anxiety symptoms and child BI scores and child BI scores were associated with child anxiety symptoms, then a mediation model was run to test whether child BI mediated the association between maternal and child symptoms.

Next, moderation analyses tested whether child BI moderated the effects of maternal anxiety on concurrent or later child anxiety. Models were fit using the PROCESS Macro (version 4.2; Hayes, 2022), with child BI entered as the moderator variable, maternal anxiety symptoms as the predictor variable, child sex as a covariate, and child anxiety symptoms as the outcome variable. Continuous predictor variables were mean-centered prior to the analyses. The interaction term in each moderation model (STAI*BI) tested for a moderation effect, with follow-up conditional effects reported at −1SD, Mean, and +1SD. Floodlight analyses were conducted to identify the Johnson-Neyman point, i.e., the value of the moderator at which the association between the predictor and outcome variables becomes significant. For interpretability, predicted values were back-transformed and plotted on the raw scale by re-adding the sample means to the mean-centered predictors.

Secondary analyses tested for evidence of sex-specific effects and for specificity of the models to anxiety. For analyses exploring anxiety specificity, models tested whether any significant findings from the main analyses remained significant when (a) testing maternal depressive symptoms in place of maternal anxiety symptoms and (b) testing child externalizing symptoms in place of child anxiety symptoms.

## Results

### Sample characteristics

Table 1 displays the sample sociodemographic characteristics. Children were predominantly non-Hispanic White (72%) and of middle- to high-socioeconomic status as reflected in maternal education and annual household income. All children were born full term. Children were also of expected birthweight for gestational age (*M* = 3495 g, *SD* = 493 g, 98% > 2500 g). As noted above, children were excluded from the study if there were any known neurological disorder or trauma, developmental delays, autism spectrum disorder diagnosis, and/or maternal use of medication during pregnancy that may have significant impact on fetal brain development.

**Table 1.** Sample characteristics (*N* = 541)

	*M (SD)*	*Range*	*N (%)*
Child race			
White	–	–	429 (79)
Asian	–	–	15 (3)
Black or African American	–	–	11 (2)
Multi-racial	–	–	77 (14)
Missing	–	–	9 (2)
Child ethnicity			
Non-Hispanic/Latinx	–	–	485 (90)
Hispanic/Latinx	–	–	50 (9)
Missing	–	–	6 (1)
Child sex			
Male	–	–	290 (54)
Female	–	–	251 (46)
Maternal educational attainment			
High School/GED	–	–	22 (4)
Associate Degree	–	–	7 (1)
Bachelor’s Degree	–	–	156 (29)
Master’s Degree	–	–	245 (45)
M.D., Ph.D., J.D. or equivalent	–	–	108 (20)
Missing	–	–	3 (0.6)
Annual household income			
$5,000−$34,999	–	–	15 (3)
$35,000−$49,999	–	–	18 (3)
$50,000−$74,999	–	–	51 (9)
$75,000−$99,999	–	–	79 (15)
$100,000+	–	–	336 (62)
Missing	–	–	42 (8)
Maternal anxiety symptoms, infancy (STAI-T)	33.8 (7.9)	20−64	–
Maternal anxiety symptoms, 3y (STAI-T)	33.1 (7.5)	21−59	–
Child behavioral inhibition, 3y	0.1 (0.7)	−1.4−2.1	–
Child anxiety symptoms, 3y (ITSEA)^a^	0.0 (0.8)	−1.4−4.5	–
Child anxiety symptoms, 5y (CBCL)^b^	2.8 (2.7)	0−13	–
Maternal depressive symptoms, infancy (BDI-IA)	5.7 (4.1)	0−27	–
Maternal depressive symptoms, 3 years (BDI-IA)	5.3 (4.5)	0–40	–
Child externalizing *T*-score, 3 years (ITSEA)	48.0 (7.1)	34–75	–
Child externalizing *T*-score, 5 years (CBCL)	43.3 (9.5)	28−85	–

*Note.* STAI-T = Trait Anxiety form of the Spielberger State–Trait Anxiety Inventory; ITSEA = Infant–Toddler Social and Emotional Assessment; CBCL = Child Behavior Checklist 1½-5. BDI-IA = Revised Beck Depression Inventory.

a Child anxiety symptoms score at 3 years is the mean score of the standardized Anxiety/Worry and Separation Distress scales from the ITSEA.

b Child anxiety symptoms score at 5 years is the raw score of the Anxiety Problems DSM-Oriented scale from the CBCL 1½-5.

### Descriptive analyses

Relevant data were available for 541 participants, with questionnaire data available for 679 participants at the infancy assessment, 456 at the 3-year assessment, and 426 at the 5-year assessment. BI data from the 3-year assessment were available for 364 participants. Participants missing data at 3 years and/or 5 years did not differ on maternal anxiety symptoms at infancy. Analyses were conducted using all available data for each model.

Table 1 includes the descriptive data for the main study variables. Table 2 presents the bivariate correlations among the main study variables. As shown in Table 2, maternal anxiety symptoms were relatively stable from infancy to 3 years and were consistently positively correlated with child anxiety symptoms at ages 3 years and 5 years. Child BI at age 3 years was not correlated with maternal anxiety symptoms at either infancy or 3 years. Greater child BI at 3 years was correlated with greater child anxiety symptoms at 3 years but not at 5 years. The child anxiety scores were moderately correlated from 3 years to 5 years. *T*-test analyses revealed sex differences for child anxiety symptoms at 3 years, *t* (442) = 2.79, *p* = .006, with females exhibiting greater symptoms than males; sex differences at 5 years approached significance, *t* (421) = 1.76, *p* = .080, with females tending to exhibit more symptoms. There were no observed sex differences for child BI scores at 3 years, *t* (360) = 0.72, *p* = .474. Given the observed sex differences in anxiety symptoms, sex was included as a covariate in subsequent analyses.

**Table 2.** Correlations among main study variables

	STAI-T (infancy)	STAI-T (3 years)	Child BI (3 years)	ITSEA-ANX (3 years)
STAI-T (3 years)	0.72***	–	–	–
Child BI (3 years)	0.01	0.00	–	–
ITSEA-ANX (3 years)	0.15**	0.21***	0.15**	–
CBCL-ANX (5 years)	0.18***	0.23***	0.06	0.42***

*Note.* STAI-T = Trait Anxiety form of the Spielberger State-Trait Anxiety Inventory; BI = behavioral inhibition; ITSEA-ANX = mean score of standardized Anxiety/Worry and Separation Distress scales from the Infant-Toddler Social and Emotional Assessment; CBCL-ANX = Anxiety Problems DSM-oriented scale from the Child Behavior Checklist 1½-5.

** *p* < .01, ***p < .001.

### Mediation analyses

Correlation analyses did not support potential mediation effects: Although maternal anxiety symptoms at infancy and 3 years were associated with child anxiety symptoms at 3 years and 5 years and child BI scores were associated with child anxiety symptoms at 3 years, child BI was not associated with earlier or concurrent maternal anxiety scores. Therefore, for the sake of parsimony, mediation models were not run to test whether child BI mediated associations between maternal and child symptoms.

### Moderation analyses

**Figure 2.**
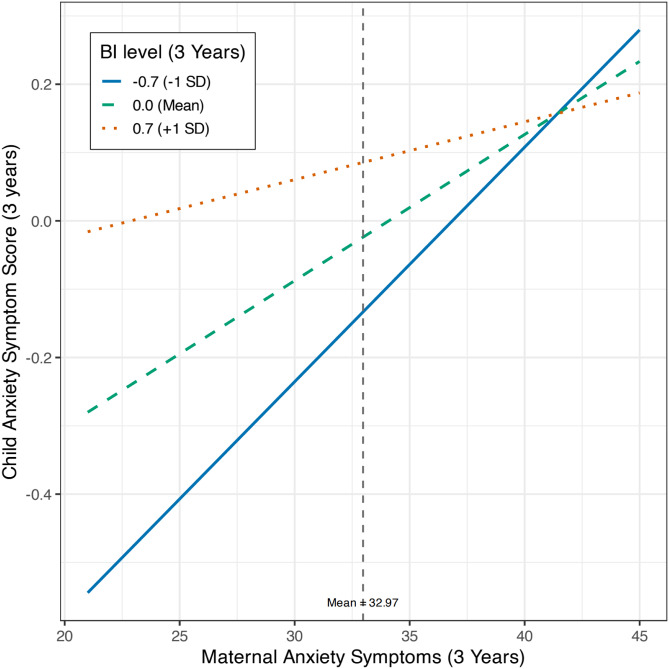
Moderating effect of child behavioral inhibition (BI) on the association between maternal anxiety symptoms and child anxiety symptoms at age 3 years. Conditional effects at −1SD, mean, +1SD. Association between maternal anxiety symptoms and child anxiety symptoms significant at −1SD and mean but not at +1SD of child BI.

**Figure 3.**
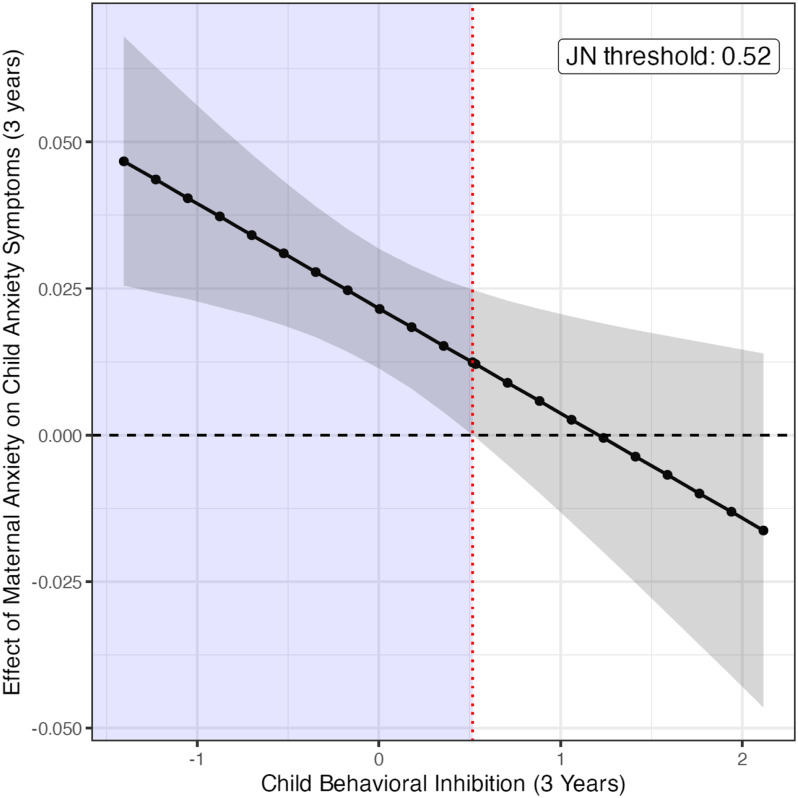
Floodlight analysis for the moderating effect of child behavioral inhibition on the association between maternal anxiety symptoms and child anxiety symptoms at age 3 years. Johnson-Neyman point denotes the value of child behavioral inhibition at which the association between maternal anxiety symptoms and child anxiety symptoms becomes significant.

We ran two moderation models to test whether child BI moderated the association between maternal anxiety symptoms and child anxiety symptoms. In the first model, we examined concurrent associations, i.e., we tested whether the effect of the interaction between child BI at 3 years and maternal anxiety symptoms at 3 years on child anxiety symptoms at 3 years was significant (Table 3). The overall model was significant, *F*(4, 351) = 9.88, *p* < .001, *R* ^2^ = .10. There was a positive association between maternal anxiety symptoms and child anxiety symptoms, *β* = 0.21, *p* < .001, and between child BI and child anxiety symptoms, *β* = 0.14, *p* = .005. Additionally, the interaction term between maternal anxiety symptoms and child BI (*β* = −0.13, *p* = .009) supported a moderation effect. As displayed in Table 4 and Figure 2, the magnitude of the positive association between maternal anxiety symptoms and child anxiety symptoms increased as the level of child BI decreased. That is, the association between maternal and child symptoms was more robust among children with lower BI levels. Children with higher BI levels had consistently higher levels of anxiety symptoms, regardless of the level of maternal anxiety symptoms. The Johnson–Neyman value was 0.50 (0.52 on the raw, uncentered BI scale shown in Figure 2), which corresponded to a significance region for children with BI scores at or below the 78th percentile in our sample. For children with BI scores above this threshold (top 22% of the sample), the association between maternal and child symptoms was not significant. Floodlight analysis of these findings is plotted in Figure 3.

**Table 3.** Regression coefficients for the concurrent moderation model predicting child anxiety symptoms at 3 years

Predictor	*b*	*β*	SE	*t*	*p*	LLCI	ULCI
Maternal anxiety symptoms (3 years)	0.02	0.21	0.01	4.13	<.001	0.011	0.032
Child behavioral inhibition (3 years)	0.15	0.14	0.05	2.80	.005	0.045	0.258
Maternal anxiety × Child behavioral inhibition	−0.02	−0.13	0.01	−2.63	.009	−0.031	−0.005
Child sex	0.17	0.23	0.08	2.21	.028	0.019	0.328

**Table 4.** Conditional effects of maternal anxiety at values of child behavioral inhibition (−1 SD, mean, +1 SD) for the concurrent moderation model predicting child anxiety symptoms at 3 years

Child behavioral inhibition	*b*	*β*	SE	*t*	*p*	LLCI	ULCI
−0.722	0.034	0.34	0.01	4.87	<.001	0.021	0.048
0.000	0.021	0.21	0.01	4.12	<.001	0.011	0.032
0.722	0.009	0.08	0.01	1.17	.242	−0.006	0.023

**Figure 4.**
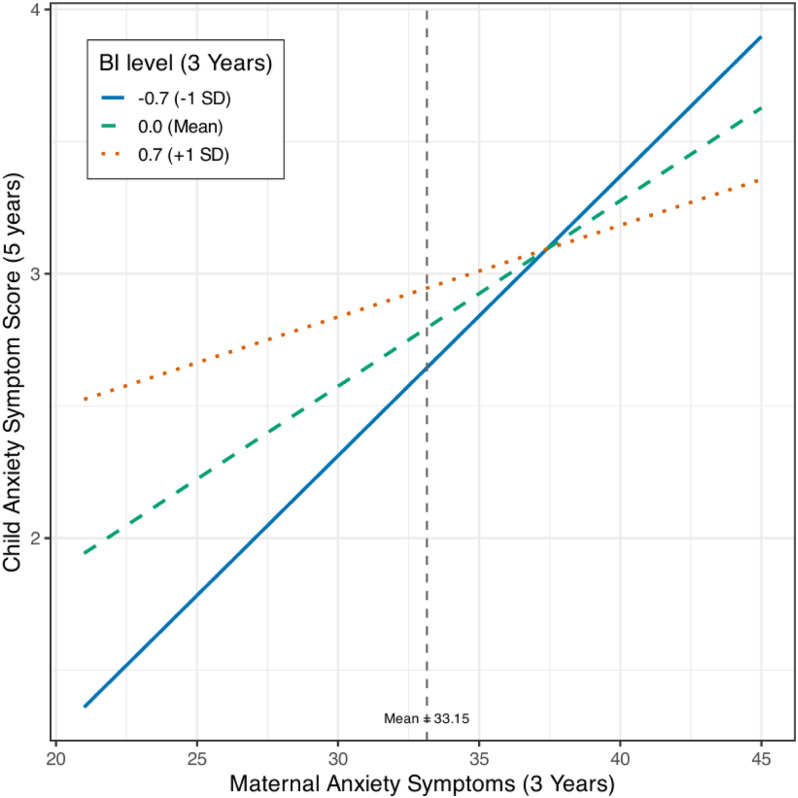
Moderating effect of child behavioral inhibition (BI) on the association between maternal anxiety symptoms and child anxiety symptoms at age 5 years. Conditional effects at −1SD, mean, +1SD. Association between maternal anxiety symptoms and child anxiety symptoms significant at −1SD and mean but not at +1SD of child BI.

**Figure 5.**
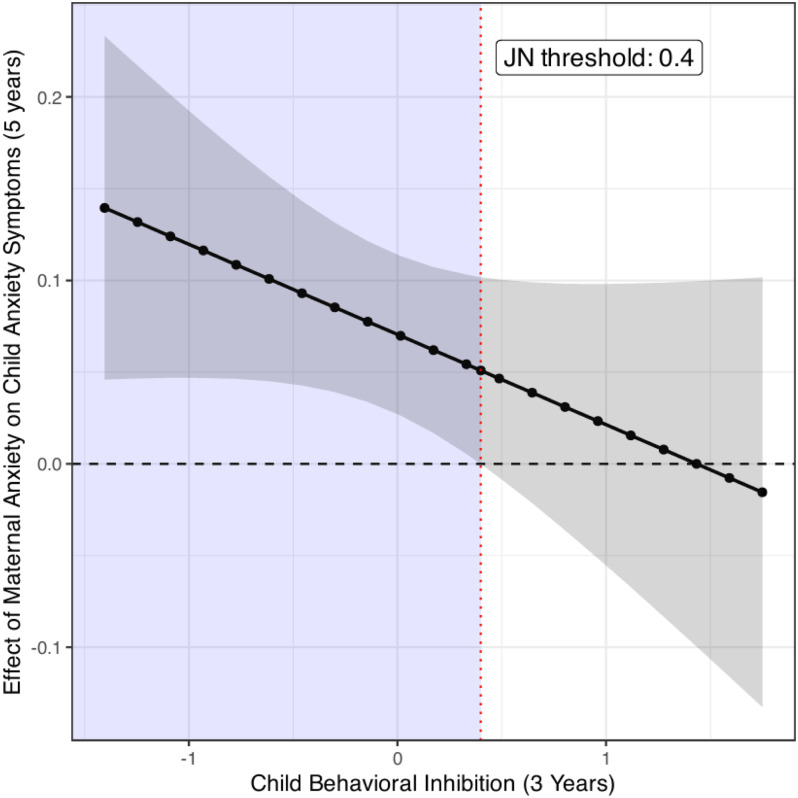
Floodlight analysis for the moderating effect of child behavioral inhibition on the association between maternal anxiety symptoms and child anxiety symptoms at age 5 years. Johnson-Neyman point denotes the value of child behavioral inhibition at which the association between maternal anxiety symptoms and child anxiety symptoms becomes significant.

In the second model, we examined predictive associations, i.e., we tested whether the effect of the interaction between child BI at 3 years and maternal anxiety symptoms at 3 years on child anxiety symptoms at 5 years was significant. The overall model was significant, *F*(4, 267) = 4.35, *p* = .002, *R* ^2^ = .06. There was a positive association between maternal anxiety symptoms and child anxiety symptoms, *β* = 0.19, *p* = .002. The association between child BI and child anxiety symptoms was not significant, *β* = 0.05, *p* = .362. The interaction term between maternal anxiety symptoms and child BI (*β* = −0.10, *p* = .113) suggested a moderation effect was not observed according to traditional statistical significance criteria; however, the effect size was similar to that observed for the concurrent model at age 3 years. Table 5 displays the full model results. Table 6 and Figure 4 display the conditional effects for the predictive model, which demonstrated a similar pattern of associations as observed in the concurrent model at age 3 years. The Johnson–Neyman value was 0.39, which corresponded to a significance region for children with BI scores at or below the 76th percentile in our sample. For children with BI scores above this threshold (top 24% of the sample), the association between maternal and child symptoms was not significant. This significance region was very similar to that observed at age 3 years (Figure 5).

**Table 5.** Regression coefficients for the predictive moderation model predicting child anxiety symptoms at 5 years

Predictor	*b*	*β*	SE	*t*	*p*	LLCI	ULCI
Maternal anxiety symptoms (3 years)	0.07	0.19	0.02	3.18	.002	0.027	0.114
Child behavioral inhibition (3 years)	0.21	0.05	0.23	0.91	.362	−0.241	0.658
Maternal anxiety × Child behavioral inhibition	−0.05	−0.10	0.03	−1.59	.113	−0.110	0.012
Child sex	0.45	0.16	0.33	1.36	.174	−0.200	1.102

**Table 6.** Conditional effects of maternal anxiety at values of child behavioral inhibition (−1 SD,mean, +1 SD) for the predictive moderation model predicting child anxiety symptoms at 5 years

Child behavioral inhibition	*b*	*β*	SE	*t*	*p*	LLCI	ULCI
−0.717	0.11	0.29	0.03	3.49	.001	0.046	0.165
0.000	0.07	0.19	0.02	3.18	.002	0.027	0.114
0.717	0.04	0.09	0.03	1.08	.280	−0.029	0.098

### Secondary analyses

#### Sex-specific effects

Because sex-specific effects may be obscured when testing at the level of the entire sample, all mediation and moderation analyses were considered. As with the analyses of the entire sample, sex-specific effects for mediation models were only tested if there was support in correlation analyses. As with the sample as a whole, BI was not associated with earlier or concurrent maternal anxiety scores among males or among females, *ps* ≥ .42. Therefore, mediational models were not tested.

Next, the two moderation models described above (concurrent, predictive) were run with the inclusion of the two 2-way (STAI*sex, BI*sex) and one 3-way (STAI*BI*sex) sex interaction terms. In the predictive model only, the sex*STAI interaction term was significant; follow-up analyses suggested that the positive association between maternal anxiety symptoms at 3 years and child anxiety symptoms at 5 years was stronger among males (*r* = .33, *p* < .001) than among females (*r* = .10, *p* = .231), *Z*-test statistic = 2.24, *p* = .012. None of the 2-way or 3-way interaction terms involving sex and BI were significant in either the concurrent or predictive model; thus, there was no evidence for sex-specific effects of BI on child anxiety in either of the moderation models. Supplementary Tables 1 and 2 present the full moderation model results.

#### Specificity to anxiety

To examine whether the significant moderation findings in the main analyses were specific to anxiety, the concurrent moderation model was run twice. In the first model, maternal depressive symptoms were used in place of maternal anxiety symptoms (i.e., BDI at 3 years in place of maternal STAI at 3 years). The overall model was significant, *F*(4, 351) = 6.80, *p* < .001, R^2^ = .07, as were the main effects for maternal depressive symptoms at age 3 years and child BI at age 3 years in predicting child anxiety symptoms at age 3 years. However, the interaction term (BDI*BI) was not significant, *β* = −0.09, *p* = .161, indicating a lack of moderation of the effects of maternal depressive symptoms on child anxiety symptoms by child BI. Supplementary Table 3 presents the full model results.

In the second model, maternal anxiety symptoms at 3 years, child BI at 3 years, and the interaction between these variables were included as predictors of child externalizing symptoms at 3 years. For this model, the overall model was significant, *F*(4, 338) = 7.72, *p* < .001, R^2^ = .08, as was the main effect for maternal anxiety symptoms at age 3 years, *β* = 0.27, *p* < .001. However, neither the main effect of child BI, *β* = −0.06, *p* = .278, nor the interaction term (STAI*BI) was significant, *β* = −0.05, *p* = .360, supporting the specificity of BI to child anxiety symptoms. Supplementary Table 4 presents the full model results.

## Discussion

The intergenerational transmission of anxiety is well documented, with children of parents with an anxiety disorder at significantly increased risk for developing an anxiety disorder themselves. Understanding the responsible mechanisms involved in this intergenerational transmission is of high public health importance given that anxiety is the most common mental health condition affecting children and adults. In addition, anxiety can be refractory to treatment and have a negative impact across a range of domains of functioning across the lifespan. In this study, we focused on the potential role of child BI in the intergenerational transmission of anxiety, given that child BI has been linked to both parental history of anxiety and to the development of child anxiety. Specifically, we examined the role of child BI as a potential mediator and/or moderator of the effects of maternal anxiety on child anxiety in the first five years of life. We did not find evidence for mediation effects, but we did observe moderation effects: Children with moderate to low BI showed more robust associations between maternal and child symptoms than children with high BI; children with high BI demonstrated elevated symptoms regardless of maternal symptom level. We examined both concurrent and longitudinal associations and found similar effect sizes in the concurrent and predictive models, with the interaction effect reaching statistical significance in the concurrent model. These findings showed specificity, with child BI associated only with child anxiety symptoms and not with child externalizing symptoms. Further, only maternal anxiety symptoms and not maternal depressive symptoms interacted with child BI to predict child anxiety symptoms. Finally, analysis for potential sex-specific effects found that female children showed higher levels of anxiety symptoms than male children and that the association between maternal and child anxiety symptoms were more robust among male children than female children; however, there were no sex-specific effects in the pattern of associations involving child BI.

The current study did not find evidence that child BI mediated the association between maternal and child symptoms. Although maternal anxiety symptoms at infancy and age 3 years were associated with child anxiety symptoms at ages 3 years and 5 years, maternal symptoms were not associated with child BI at 3 years. Previous studies have linked parental anxiety to increased likelihood of BI in offspring (Aktar & Pérez-Edgar, 2024; Fox et al., 2023; Rosenbaum et al., 1993). However, these studies have primarily focused on clinical levels of anxiety, i.e., anxiety disorders, in parents, whereas this study examined continuous measures of trait anxiety symptoms. Notably, there is some evidence that parental history of anxiety, regardless of current diagnostic status, is predictive of child BI, suggesting that even if parents’ anxiety has remitted at the time of assessment, their children continue to be at increased likelihood of being behaviorally inhibited (Murray et al., 2014). Thus, parental lifetime diagnostic history may be more predictive of associations between parental anxiety and child BI. Additionally, the literature is mixed as to whether the specific nature of the parent’s anxiety disorder is relevant for child BI. For example, some studies suggest that BI is particularly relevant in relation to social anxiety disorder, whereas others have found that various parental anxiety disorders (e.g., generalized anxiety, panic disorder, agoraphobia) are associated with child BI (Fox et al., 2023; Hudson et al., 2019; Rosenbaum et al., 1993). More research is needed to determine the genetic, biological, and environmental factors that increase the likelihood that a child will exhibit behaviorally inhibited tendencies in early life, as to date there is limited research on the precursors of child BI (Mudra et al., 2022).

In the current study, we found evidence for a moderation effect of child BI on the association between maternal and child symptoms, but the nature of the effect was not as hypothesized. Based on the admittedly inconsistent literature to date, we hypothesized that the effect of maternal anxiety on child anxiety would be potentiated among children with high BI. Instead, we found that the association between maternal and child symptoms was greatest in magnitude among children with the lowest BI levels. An interrogation of the interaction effect showed that the association between maternal and child symptoms was not significant among children with the highest BI levels. Rather, children with the highest BI scores had elevated anxiety symptoms, regardless of maternal anxiety symptom level. Among children with moderate to low levels of BI, greater maternal anxiety symptoms were associated with greater child anxiety symptoms. This pattern of findings aligns with hypotheses that suggest that exposure to maternal anxiety may only increase anxiety risk among children with low BI, as children with high BI already exhibit heightened risk that is not further increased by the presence of maternal anxiety (Aktar & Pérez-Edgar, 2024). Our findings are also consistent with suggestions that BI and parental anxiety share or influence similar underlying biological mechanisms that affect child anxiety vulnerability, such that having one or both risk factors imparts similar child risk (Fox et al., 2023). That is, our findings are aligned with those that have found that children with high BI do not demonstrate increased risk in the context of parental anxiety, whereas children who are not temperamentally at risk are more affected by parental anxiety (Aktar & Pérez-Edgar, 2024).

The moderation effect (i.e., the interaction term between maternal anxiety at 3 years and child BI at 3 years) met statistical significance in the concurrent model predicting child anxiety at age 3 years, but not in the longitudinal model predicting child anxiety at age 5 years. Open questions in the child BI literature include (a) how early in development observations of BI can predict anxiety and (b) how far into development BI assessed in early childhood can predict later anxiety. Although some studies have documented associations between child BI assessed in early childhood and anxiety in later childhood, adolescence, or adulthood, other studies have failed to find such associations (Lawrence et al., 2020; Lorenzo et al., 2022; Tang et al., 2020; Zeytinoglu et al., 2025). In the current study, BI was assessed at age 3 years and found to be associated with concurrent child anxiety symptoms, both in correlation analyses and in regression analyses as a main effect and as an interactive effect with maternal anxiety symptoms. In longitudinal analyses, child BI at 3 years was not associated with child anxiety symptoms at 5 years in correlation analyses or as a main effect in regression analyses. Further, the interaction term between child BI and maternal anxiety symptoms at age 3 years did not reach statistical significance in predicting child anxiety symptoms at 5 years. However, the effect size for the interaction term was similar to that at age 3 years, and the follow-up test of conditional effects demonstrated a very similar pattern of results to those observed at age 3 years. There are a number of possible reasons that the patterns may have been similar but potentially more robust for the concurrent model. First, the somewhat smaller sample size at age 5 years relative to age 3 years may have influenced the relative power to detect moderation effects in the predictive model versus the concurrent model. Second, different measures were used to assess child anxiety symptoms at 3 years (ITSEA) and 5 years (CBCL 1.5–5). Although the two scales assess similar constructs (generalized anxiety/worry, phobias, separation anxiety), any differences between the items comprising the scales may have contributed to differences in findings. Further, associations between BI and varied anxiety symptoms (e.g., total anxiety symptom scales including symptoms across anxiety phenotypes) may become weaker with age as BI becomes more relevant for predicting specific forms of anxiety (e.g., social anxiety) and as the most commonly presenting forms of anxiety change and differentiate with development (Lorenzo et al., 2022). Additionally, the correlation between the anxiety symptom scores at 3 years and 5 years was moderate, which also may speak to differences between the scales as well as to modest stability in non-specific anxiety symptoms across this age period. Moreover, BI observed in response to novel social vs non-social stimuli may have varying predictive relevance for different anxiety conditions (e.g., social anxiety related to responses to novel social stimuli, specific phobias related to responses to novel non-social stimuli; Dyson et al., 2011; Fox et al., 2023). Nevertheless, despite these methodological considerations, the pattern of moderation that we observed across the two ages was quite similar. Research is needed to determine how BI may predict specific anxiety phenotypes across development, taking into account changes in anxiety presentation across childhood. Such an approach may further clarify the role of child BI in the developmental psychopathology of anxiety across childhood, including within the context of intergenerational processes.

Notably, BI has been found to be only modestly stable across development, and most children classified as behaviorally inhibited do not develop an anxiety disorder (Fox et al., 2023; Lorenzo et al., 2022). In the current study, we only had one timepoint (age 3 years) when we had a behavioral observation of child BI. Our findings may have been further strengthened if we had child BI assessments at multiple timepoint to provide a measure of stable BI, which evidence suggests may be a more robust predictor of later anxiety risk than assessment at a single timepoint (Chronis-Tuscano et al., 2009). Relatedly, the anxiety risk for some of the children assessed as high in BI at 3 years may have been reduced by age 5 years (e.g., due to caregiving or other contextual processes) so that they were no longer at elevated risk. There also may be sleeper effects that do not emerge until later in development. For example, adolescence is a period of significant risk for the development of anxiety (Sylvester & Luby, 2024). Associations between BI in early childhood and later anxiety may not become apparent until these youth pass through such sensitive risk periods. Conversely, some studies suggest that anxiety risk for children high in BI in early childhood diminishes throughout childhood into adolescence (Aktar & Pérez-Edgar, 2024; Hudson et al., 2019). Thus, longitudinal assessment throughout childhood and adolescence is necessary to determine the role of child BI in the intergenerational transmission of anxiety across development. Finally, a number of studies suggest that BI by itself may not be as robust a predictor of risk for anxiety as BI in combination with other individual risk factors not assessed here (e.g., heightened error monitoring, attentional biases to threat, inhibitory control; Aktar et al., 2013; Fox et al., 2023; Hudson et al., 2019; Lawrence et al., 2020; Murray et al., 2014; Tang et al., 2020). Research that includes examination of how maternal anxiety may interact with child BI and additional moderating risk factors throughout childhood and adolescence is therefore warranted.

Secondary analyses tested for sex-specific effects and for specificity of the main findings to anxiety. The extant literature suggests the potential for sex-specific effects, given documented differences in how males and females respond to maternal psychopathology in early life and differential consequences of inhibited temperament on socioemotional development. In the current analyses, there was evidence that maternal anxiety symptoms at age 3 years had a more robust association with child anxiety symptoms at age 5 years among male children compared to female children. These findings are consistent with literature suggesting that the effects of maternal psychopathology on child outcomes may differ by child sex, with the nature of the differential sex effects dependent on many factors (e.g., type of maternal psychopathology, child outcome under study, timing of exposure to maternal psychopathology, timing of assessment of child outcome). None of the other interaction terms were significant, including all interaction terms involving BI; thus, the results did not suggest differential sex effects in relation to child BI. Notably, the literature on potential sex differences in the consequences of BI for child socioemotional outcomes is mixed, and any such differences may not emerge until later in development (Rubin & Barstead, 2014). Moreover, our tests for sex-specific effects involved three-way interaction terms, and this study may have been underpowered to robustly test for such effects (Castillo et al., 2026). Further research is needed to determine when in development the various processes driving the instigation and maintenance/exacerbation of anxiety begin to diverge by sex.

To explore anxiety specificity, we followed up the significant findings of the main analyses, first examining whether child BI moderated the effects of maternal depressive symptoms on child anxiety symptoms. We did not find evidence of an interactive effect. We further examined whether child BI and maternal anxiety symptoms were associated with child externalizing problems. Although greater maternal anxiety symptoms were associated with greater child externalizing problems, neither child BI nor the interaction between child BI and maternal anxiety symptoms were associated with child externalizing problems. Together, these results support the specificity of child BI in relation to both maternal and child anxiety symptoms in intergenerational processes involved in child anxiety.

The current findings should be considered in the context of the study’s strengths and limitations. Strengths include the large sample followed longitudinally, allowing for concurrent and predictive analyses. Another strength is the use of a standardized observational measure of child behaviorally inhibited behaviors in response to a variety of tasks, coded by raters blind to other data. Many studies have relied on more limited assessments, such as parent- or teacher-report or raters assessing child behavior during non-relevant tasks (e.g., cognitive assessment); such approaches may produce less valid or robust associations (Tang et al., 2020). Another potential strength of the study is the use of a continuous measure of BI. Historically, many studies have used extreme group designs, with some conceptualizing BI as a categorical temperament dimension, with only those in the top 10% of behaviorally inhibited behaviors considered behaviorally inhibited and at increased risk for anxiety outcomes (Shamir-Essakow et al., 2005). Such extreme group approaches may obscure the role of more moderate levels of behaviorally inhibited tendencies on intergenerational anxiety processes. Notably, in the current study, the “cut point” for interactive effects between child BI and maternal anxiety symptoms on child anxiety symptoms occurred for children in the top 22% of child BI scores when predicting child anxiety at age 3 years and in the top 24% of child BI scores when predicting child anxiety at age 5 years. An additional strength of the current study is the inclusion of secondary analyses that support the specificity of the role of BI in the intergenerational transmission of anxiety (as opposed to psychopathology more generally).

A potential limitation of our BI measure is that it did not include scoring of child expressions of fear/negative affect. According to Fox et al. (2023), BI is reflected in child reactions to novel contexts, including being slow to approach, seeking proximity to caregivers, and expressing negative affect. Our BI measure incorporated assessment of child time to approach and proximity seeking of their caregiver but did not include codes for child affective expressions. Incorporation of such behaviors in the scale may improve its predictive ability. Another potential limitation is that the current study did not include parenting behaviors. Thus, we could not determine if parenting behaviors were responsible for any effects (main or interactive) of maternal anxiety symptoms on child anxiety symptoms, as suggested by the literature. The use of maternal report for assessment of both maternal and child anxiety symptoms may have inflated their associations due to issues of shared method variance and reporting bias. These concerns are somewhat tempered by the fact that the focus of this study is on the potential mediating/moderating role of child BI, which is not influenced by these issues, as the BI measure was observational and not based on maternal report. Moreover, recent work suggests that mothers’ psychopathology produces minimal bias in their ratings of their children’s symptoms (Olino et al., 2021). Finally, the current study focused on mothers and did not consider the role of fathers. Data suggest that both paternal and maternal anxiety increase child anxiety risk and that child BI may be expressed differently in interactions with fathers versus mothers in early childhood (Aktar et al., 2013, 2014). Future studies should consider the joint roles of maternal and paternal anxiety in the context of child BI and the intergenerational transmission of anxiety.

In summary, the current study explored the role of child behavioral inhibition in the intergenerational transmission of anxiety in early childhood. In this community sample of typically developing children, elevated maternal anxiety symptoms were associated with increased concurrent and later child anxiety symptoms. We did not observe evidence that child BI mediated the association between maternal and child symptoms, as maternal anxiety symptoms were not associated with child BI. There was evidence for a moderation effect: Children with high BI exhibited heightened levels of anxiety symptoms, regardless of severity of maternal anxiety symptoms. Among children with moderate to low BI, greater maternal anxiety was associated with higher levels of child anxiety symptoms. Thus, the results suggest that maternal symptoms have more impact on child symptoms when the child is not already at elevated risk due to temperamental characteristics. Moreover, these associations showed specificity to intergenerational anxiety processes (versus other forms of maternal or child psychopathology). Overall, the current findings contribute to our understanding of the role of child BI in the intergenerational transmission of anxiety in early childhood.

## Supporting information

10.1017/S0954579426101643.sm001 Bosquet Enlow et al. supplementary material Bosquet Enlow et al. supplementary material

## Data Availability

Availability of data Deidentified data sets will be made available upon reasonable request of the first author. Availability of code Syntax required to reproduce analyses will be made available upon reasonable request of the first author. We have cited all statistical programs used in these analyses. Availability of methods/materials Information about study methods and materials will be made available upon reasonable request of the first author. We have cited all measures used in these analyses. STROBE guidelines were followed as relevant.

## Supplementary material

The supplementary material for this article can be found at https://doi.org/10.1017/S0954579426101643.

## Pre-registration statement

This research was not pre-registered.
