# Supplementary material for: The role of child behavioral inhibition in the intergenerational transmission of anxiety in early childhood
Source: Dev Psychopathol. 2026 Jul 9:1–13. Online ahead of print. doi: 10.1017/S0954579426101643 (PMC13366353; doi:10.1017/S0954579426101643)
Supplement: Bosquet Enlow et al. supplementary material [file S0954579426101643sup001.docx]

Supplementary Materials

This file includes the following tables for “The Role of Child Behavioral Inhibition in the Intergenerational Transmission of Anxiety in Early Childhood”:

Supplementary Table 1. Regression coefficients for the concurrent moderation model predicting child anxiety symptoms at 3 years, including sex-interaction terms

Supplementary Table 2. Regression coefficients for the predictive moderation model predicting child anxiety symptoms at 5 years, including sex-interaction terms

Supplementary Table 3. Regression coefficients for the concurrent moderation model predicting child anxiety symptoms at 3 years, with maternal depressive symptoms as the predictor

Supplementary Table 4. Regression coefficients for the concurrent moderation model predicting child externalizing symptoms at 3 years

**Supplementary Table 1.** *Regression coefficients for the concurrent moderation model predicting child anxiety symptoms at 3 years, including sex-interaction terms*

| Predictor | *b* | β | SE | *t* | *p* | LLCI | ULCI |
| --- | --- | --- | --- | --- | --- | --- | --- |
| Maternal anxiety symptoms, 3 years | 0.02 | 0.23 | 0.01 | 3.33 | .001 | 0.010 | 0.038 |
| Child behavioral inhibition, 3 years | 0.22 | 0.20 | 0.08 | 2.79 | .005 | 0.065 | 0.371 |
| Child sex | 0.17 | 0.23 | 0.08 | 2.22 | .027 | 0.020 | 0.329 |
| Maternal anxiety x Child behavioral inhibition | -0.01 | -0.09 | 0.01 | -1.31 | .191 | -0.031 | 0.006 |
| Maternal anxiety x Child sex | -0.01 | -0.05 | 0.01 | -0.53 | .598 | -0.026 | 0.015 |
| Child behavioral inhibition x Child sex | -0.13 | -0.12 | 0.11 | -1.21 | .226 | -0.344 | 0.082 |
| Maternal anxiety x Child behavioral inhibition x Child sex | -0.01 | -0.09 | 0.01 | -0.90 | .369 | -0.039 | 0.015 |

*Note.* The overall model was significant, F(7, 348) = 6.01, p < .001, R2 = .11.

**Supplementary Table 2.** *Regression coefficients for the predictive moderation model predicting child anxiety symptoms at 5 years, including sex-interaction terms*

| Predictor | *b* | β | SE | *t* | *p* | LLCI | ULCI |
| --- | --- | --- | --- | --- | --- | --- | --- |
| Maternal anxiety symptoms, 3 years | 0.13 | 0.35 | 0.03 | 4.08 | .000 | 0.066 | 0.189 |
| Child behavioral inhibition, 3 years | 0.10 | 0.03 | 0.35 | 0.29 | .770 | -0.581 | 0.784 |
| Child sex | 0.44 | 0.16 | 0.33 | 1.33 | .185 | -0.210 | 1.082 |
| Maternal anxiety x Child behavioral inhibition | 0.01 | 0.02 | 0.05 | 0.22 | .828 | -0.088 | 0.110 |
| Maternal anxiety x Child sex | -0.11 | -0.30 | 0.04 | -2.48 | .014 | -0.197 | -0.023 |
| Child behavioral inhibition x Child sex | 0.32 | 0.08 | 0.46 | 0.70 | .488 | -0.588 | 1.229 |
| Maternal anxiety x Child behavioral inhibition x Child sex | -0.09 | -0.17 | 0.06 | -1.36 | .176 | -0.213 | 0.039 |

*Note.* The overall model was significant, F(7, 264) = 3.71, p < .001, R2 = .09. Maternal anxiety symptoms were more strongly associated with child anxiety symptoms among male children compared to female children.

**Supplementary Table 3.** *Regression coefficients for the concurrent moderation model predicting child anxiety symptoms at 3 years, with maternal depressive symptoms as the predictor*

|  | *b* | β | SE | *t* | *p* | LLCI | ULCI |
| --- | --- | --- | --- | --- | --- | --- | --- |
| Maternal depressive symptoms, 3 years | 0.02 | 0.15 | 0.01 | 2.86 | .004 | 0.008 | 0.042 |
| Child behavioral inhibition, 3 years | 0.15 | 0.14 | 0.06 | 2.75 | .006 | 0.043 | 0.260 |
| Maternal depressive symptoms x Child behavioral inhibition | -0.02 | -0.09 | 0.01 | -1.41 | .161 | -0.049 | 0.008 |
| Child sex | 0.18 | 0.24 | 0.08 | 2.27 | .024 | 0.024 | 0.338 |

**Supplementary Table 4.** *Regression coefficients for the concurrent moderation model predicting child externalizing symptoms at 3 years*

|  | *b* | β | SE | *t* | *p* | LLCI | ULCI |
| --- | --- | --- | --- | --- | --- | --- | --- |
| Maternal anxiety symptoms | 0.25 | 0.27 | 0.05 | 5.08 | 0.000 | 0.151 | 0.343 |
| Child behavioral inhibition | -0.56 | -0.06 | 0.52 | -1.09 | 0.278 | -1.577 | 0.455 |
| Maternal anxiety x Child behavioral inhibition | -0.06 | -0.05 | 0.06 | -0.92 | 0.360 | -0.185 | 0.068 |
| Child sex | -1.52 | -0.22 | 0.74 | -2.07 | 0.040 | -2.976 | -0.074 |
